# Insulin-Producing Cells Differentiated from Human Bone Marrow Mesenchymal Stem Cells *In Vitro* Ameliorate Streptozotocin-Induced Diabetic Hyperglycemia

**DOI:** 10.1371/journal.pone.0145838

**Published:** 2016-01-12

**Authors:** Ying Xin, Xin Jiang, Yishu Wang, Xuejin Su, Meiyu Sun, Lihong Zhang, Yi Tan, Kupper A. Wintergerst, Yan Li, Yulin Li

**Affiliations:** 1 Key Laboratory of Pathobiology, Ministry of Education, Jilin University, Changchun, China; 2 Department of Radiation Oncology, The First Hospital of Jilin University, Changchun, China; 3 Department of Pediatrics, Division of Endocrinology, University of Louisville, Wendy L. Novak Diabetes Care Center, Louisville, Kentucky, United States of America; 4 Department of Orthopedic Surgery, Karolinska University Hospital, Stockholm, Sweden; Children's Hospital Boston/Harvard Medical School, UNITED STATES

## Abstract

**Background:**

The two major obstacles in the successful transplantation of islets for diabetes treatment are inadequate supply of insulin-producing tissue and immune rejection. Induction of the differentiation of human bone marrow-derived mesenchymal stem cells (hMSCs) into insulin-producing cells (IPCs) for autologous transplantation may alleviate those limitations.

**Methods:**

hMSCs were isolated and induced to differentiate into IPCs through a three-stage differentiation protocol in a defined media with high glucose, nicotinamide, and exendin-4. The physiological characteristics and functions of IPCs were then evaluated. Next, about 3 × 10^6^ differentiated cells were transplanted into the renal sub-capsular space of streptozotocin (STZ)-induced diabetic nude mice. Graft survival and function were assessed by immunohistochemistry, TUNEL staining and measurements of blood glucose levels in the mice.

**Results:**

The differentiated IPCs were characterized by Dithizone (DTZ) positive staining, expression of pancreatic β-cell markers, and human insulin secretion in response to glucose stimulation. Moreover, 43% of the IPCs showed L-type Ca^2+^ channel activity and similar changes in intracellular Ca^2+^ in response to glucose stimulation as that seen in pancreatic β-cells in the process of glucose-stimulated insulin secretion. Transplantation of functional IPCs into the renal subcapsular space of STZ-induced diabetic nude mice ameliorated the hyperglycemia. Immunofluorescence staining revealed that transplanted IPCs sustainably expressed insulin, c-peptide, and PDX-1 without apparent apoptosis *in vivo*.

**Conclusions:**

IPCs derived from hMSCs *in vitro* can ameliorate STZ-induced diabetic hyperglycemia, which indicates that these hMSCs may be a promising approach to overcome the limitations of islet transplantation.

## Introduction

Diabetes mellitus is a widespread devastating disease affecting millions of people worldwide. Although maintaining long-term glycemic control with exogenous insulin imposes an enormous physical, psychological, and financial burden on patients, it remains the only option in the face of the serious, life-threatening potential complications of diabetes. Islet transplantation is an ideal and effective treatment for type 1 diabetes; however, its application in clinical care has been largely limited by immune rejection and the shortage of donor islets [1].

Recent progress in the field of regenerative therapies has focused on the generation of surrogate β-cells from embryo-, umbilical cord blood- and various adult tissue-derived stem cells [2]. Embryonic stem cells (ESCs) can be differentiated into any cell type including insulin-producing cells (IPCs) [3]. IPCs can also be obtained by directed molecular engineering of umbilical cord blood stem cells, pancreatic stem cells, and liver stem/progenitor cells [4–6]. However, therapeutic results with the use of ESCs are not satisfactory due to a variety of challenges such as immune rejection, tumor formation, source limitations, and ethical concerns.

Recent studies have shown that mesenchymal stem cells (MSCs) have the ability to differentiate into mesenchymal, endodermal and ectodermal lineages to produce osteoblasts, adipocytes, myoblasts, and endocrine cells [7]. Transplantation of autologous MSCs would help overcome the major limitations of inadequate supply and/or allogeneic rejection. Moreover, MSCs have been shown to have an immunomodulatory effect on the suppression of the immune response in autoimmune and inflammatory diseases [8, 9]. Co-transplantation of autologous MSCs delays islet allograft rejection and generates a local immunoprivileged site for graft survival [10]. Consequently, MSCs emerge as a much better source for the generation of surrogate β-cells [11, 12].

MSCs can be isolated from many tissues including the muscle, umbilical cord blood, adipose tissue, and bone marrow. Among these, bone marrow-derived MSCs have the highest proliferation capacity and retain their pluripotency even after 50 passages [13, 14]. Currently, there are two methods commonly used to induce MSC differentiation into IPCs *in vitro*. One is to introduce key transcription regulatory factors, such as pancreatic and duodenal homeobox 1 (PDX-1) and β-cell transactivator 2 (Beta2), by genetic engineering to modulate gene expression. The other is to supply specific soluble inducers or small molecule compounds in cell culture medium to initiate and promote β-cell differentiation [15–17]. The efficiency and extent of stem cell differentiation are modulated by the internal genetic program and the external microenvironment. Due to accumulating evidence suggesting that the microenvironment plays an important role in the survival and differentiation of stem cells, understanding how to set up a microenvironment that mimics the pancreatic developmental stages and cellular differentiation is critical for successfully manipulating the differentiation of bone marrow-derived MSCs into IPCs. However, currently available protocols can only induce the differentiation of 5–10% of the MSCs into IPCs under *in vitro* conditions. Thus, the existing induction strategy needs to be modified and improved, especially since most of the studies mentioned above are based on rodent models [18, 19]. In fact, the differentiation of human MSCs into IPCs and their function of rescuing diabetes have been rarely reported [16, 20, 21].

Therefore, the present study is designed to generate well-characterized IPCs from human bone marrow-derived MSCs (hMSCs) by using a three-stage protocol and to test their potential for controlling glucose levels in diabetic mice. This study will provide evidence to support the use of adult stem cells as a steady and renewable source of IPCs for transplantation in patients with type 1 diabetes.

## Materials and Methods

### Isolation and culture of hMSCs and induction to IPCs

The protocol used in this study was approved by the Ethics Committee of the College of Basic Medical Sciences of Jilin University. Written informed consent was obtained from healthy volunteers. Human bone marrow samples were obtained from healthy volunteers by lumbar puncture in the First Hospital of Jilin University. The hMSCs were isolated and cultured as previously described [14]. Briefly, bone mononuclear cells were isolated from human bone marrow by density gradient centrifugation in a Percoll solution (1.073 g/ml, Pharmacia, USA). The cells were cultured in low glucose (5.6 mmol/L)-Dulbecco’s modified Eagle’s medium (L-DMEM) with 10% fetal bovine serum (FBS, Invitrogen, Carlsbad, CA), and the non-adherent cells were washed off after 48 h. For 8–12 days, individual colonies were selected, trypsinized, and re-plated as first-passage culture (P1).

HMSCs at passage 5 (P5) were induced to differentiate into IPCs by a three-stage protocol. In Stage 1, 2 × 10^5^ hMSCs were cultured in a 10 cm culture dish with complete medium for 12 h and then were cultured in high glucose (23.3 mmol/L)-DMEM with 5% FBS for 15 days; these cells were referred to as HD-hMSCs. Stage 2: HD-hMSCs were then cultured in L-DMEM medium containing 5% FBS and 20 μmol/L nicotinamide (Sigma, St. Louis, MO) for 7 days. Stage 3: Exendix-4 (Sigma) at 10 μmol/L was added into the medium of stage 2 culture for another 7-day incubation. The medium in every stage was replaced every 3 days.

### Adipogenic, osteogenic, and chondrogenic differentiation

To detect multi-lineage differentiation potential, P5 hMSCs were cultured in adipogenic, osteogenic, or chondrogenic medium for 2–4 weeks as previously described [22, 23]. At the end of the culture time, cells were fixed in 4% paraformaldehyde and stained with Oil red-O solution to visualize lipid droplets in the induced cells, with Von Kossa staining to evaluate the osteogenic differentiation and with Alcian blue staining to evaluate chondrogenic differentiation.

### Flow cytometry analysis

The cells were harvested, washed with phosphate buffered saline (PBS) and incubated for 1 h at 4°C with the following monoclonal antibodies (diluted at 1:100): CD73, CD166 (BD Biosciences, Bedford, MA), CD105, CD31, CD44, CD34, CD45 (Neo Marker, Fremont, CA), insulin and c-peptide (Cell signaling, Boston, MA), and then incubated with secondary antibodies of CY3 or FITC (Abcam, Cambridge, MA) for 30 min at 4°C. After washed, the cells were suspended in PBS and analyzed by FACS Calibur (BD Biosciences).

For cell cycle analysis, 1 × 10^7^ hMSCs were fixed in 70% ethanol, and stained with 50 μg/ml propidium iodide (PI, BD Biosciences) at 4°C for 30 min. DNA content was analyzed by FACS Calibur using Cell Quest software in 24 h.

### Transmission electron microscopy

1 × 10^7^ hMSCs were harvested and centrifuged to get the cells pellet. The pellet was pre-fixed in 4% glutaraldehyde, then fixed in 1% osmium tetroxide at 4°C and further dehydrated in acetone and embedded in epoxy resin. Conventional ultrathin sections were prepared. After double-staining with uranylacetate and lead citrate, sections were observed and photographed under transmission electron microscope (JEM-1200EX, JEOL Ltd).

### Karyotype

Metaphase chromosome spreads were prepared from P5 hMSCs in the exponential phase of growth. Colcemid was added to the cultures for arresting the cells in metaphase. Then the cells were trypsinized and fixed with Carnoy. Wright’s stain was used to stain chromosomes. Twenty metaphase spreads per sample were captured and karyotyped using an automated imaging system (Cytovision, Applied Imaging Corporation).

### DTZ staining

DTZ (Sigma) stock solution was prepared by dissolving 100 mg of DTZ in 5 ml of DMSO. The cells were washed with PBS and stained with 10 μL DTZ stock in 1 mL PBS solution at 37°C for 15 min. The crimson-red-stained clusters were examined under a phase-contrast microscope.

### RT-PCR

The fetal pancreas tissue from 6-month-old aborted fetus was used as the positive control. The 6-month-old fetus was obtained from a healthy pregnant volunteer by induced labor surgery in the First Hospital of Jilin University. Written informed consent was obtained from the healthy volunteer of termination of pregnancy. The protocol used in this study was approved by the Ethics Committee of the College of Basic Medical Sciences of Jilin University.

Total RNA was extracted from cells and fetal pancreas with Trizol reagent (Invitrogen). Total RNA (1 μg) was converted to cDNA using RNA PCR kit (Promega, Madison, WI). Then 2 μl of the cDNA was amplified in 20 μl system including 20 pmol of each primer pair, 10 μl of 2 × Taq PCR Master Mix (QIAGEN, Valencia, CA) and nuclease-free water.

The real-time PCR reactions were carried out using Brilliant II SYBRs Green PCR Master Mix (Agilent Technologies, Santa Clara, CA). The primer sequences were as follows: GAPDH, 5’-agaaggctggggctcatttg-3’ and 5’-aggggccatccacagtcttc-3’; Insulin, 5’-aaccaacacctgtgcggctca-3’ and 5’-tgcctgcgggctgcgtcta-3’; Ngn3, 5’-cgccggtagaaaggatgac-3’ and 5’-gagttgaggttgtgcattcg-3’; Nkx6.1, 5’-ctggagaagactttcgaacaa-3’ and 5’-agaggcttattgtagtcgtcg-3’; Pax4, 5’-attcagtggcccgtggaaa-3’ and 5’-tctcttgccgacgccattt-3’; Pdx-1, 5’-ttccggaagaaaaagagcca-3’ and 5’-aaacaggtcccaaggtggagt-3’; Glut2, 5’-cagctggccatcgtcgtcacggg-3’ and 5’-ggctcgcacaccagacaggc-3’; Glucagon, 5’-acattgccaaacgtcacgatg-3’ and 5’-gcaatgaattccttggcagct-3’. Real-time PCR was performed as the following cycling conditions: 95°C for 30 s, 60°C for 30 s, and 72°C for 60 s for 40 cycles. Comparative cycle time (CT) was used to determine fold differences between samples and normalized to an endogenous reference (GAPDH).

### Western blot assay

The cells were scraped and homogenized in RIPA buffer (Santa Cruz, Dallas, TX). The fetal pancreas tissue from 6-month-old aborted fetus was used as the positive control. Total proteins were extracted and separated on 10% SDS-PAGE gels followed by transference to a nitrocellulose membrane (Bio-Rad, Hercules, CA). The membrane was blocked with a 5% non-fat dried milk, incubated overnight at 4°C with the antibodies of anti-insulin, anti-PDX-1 and anti-β-actin (1:2000, cell signaling), and then reacted with specific secondary antibodies. The results were visualized using an enhanced chemiluminescence kit (Thermo scientific, Pittsburgh, PA)

### Measurement of insulin secretion

The cells were washed with PBS twice and placed in 5% BSA blocking medium for 5h, then selectively incubated in secretion assay buffer (SAB) containing 5.6 mM (low) glucose or 23.3 mM (high) glucose for 2 h. Insulin levels in the suspension liquid were determined via human ultrasensitive ELISA (Mercodia, Sweden) following the manufacturer’s protocol. The insulin amount was calculated by means of the human insulin standards supplied within the kit.

### L-type Ca^2+^ channel recording in differentiation process

The whole-cell patch-clamp technique and the tyrode and pipette solution were performed as described in a previous study [24]. Nifedipine-sensitive inward Ca^2+^ currents (I_Ca.L_) were recorded by 300-ms voltage step between +10 and +60 mV from a holding potential of –50 mV (to inactivate INa). Nifedipine at 10 μM suppressed the inward currents. Compensated access resistance was regularly checked and maintained below 3MΩ. Series resistance was routinely compensated by 50–70%. Membrane currents were low-pass filtered at 3 kHz and stimulation frequency was 1 Hz.

### Intracellular Ca^2+^ levels of IPCs in response to glucose stimulation

Changes in intracellular Ca^2+^ levels were estimated with a Ca^2+^-sensitive fluorescent indicator, Fluo-3/AM, as previously described [25]. The IPCs were incubated with 5 μM Fluo-3/AM (Molecular Probes, Eugene, OR) for 30 min at 37°C in the dark, washed with Ca^2+^ free-PBS twice, and then viewed in Ca^2+^ free-PBS under a confocal microscope. Fluo-3/AM was excited at 488 nm using a krypton/argon laser. Confocal fluorescence images were recorded every 3 s for 960 s. The level of intracellular Ca^2+^ at the resting stage was measured first. After 60 s, 15 μM extracellular Ca^2+^ was added to the observation chamber, 3 mM glucose was added at 240 s, 3 mM Verapamil was added at 480 s, and 3.5 mM glucose was added again at 720 s. The changes in intracellular Ca^2+^ concentration were analyzed by Time-Lapse software in 30 randomly selected IPCs, and results were represented in a graph relating fluorescence intensity to time.

### Establishment of diabetes mellitus models and cell transplantation

hMSCs and IPCs (3 × 10^6^) were labeled with BrdU (10 μmol/L) for 24 h, harvested, and treated with fibrinogen (200 μg/ml) and thrombin (0.2 U/μl) at a volume ratio of 2:1. After an incubation time of 15 min at 37°C, cell blocks were obtained.

Twenty male BALB/C nude mice, at 9 weeks of age, were obtained from Beijing Experimental Animal Technical Co., LTD (Beijing, China). This animal study was carried out in strict accordance with the recommendations outlined in the Guide for the Care and Use of Laboratory Animals of the Chinese Medicine Institute. The protocol was approved by the Ethics Committee of the College of Basic Medical Sciences of Jilin University (Permit Number: SYSK2013-0005). Mice were housed in the Jilin University animal Center at 22°C with a 12:12-h light-dark cycle and free access to rodent chow and tap water. All mice were kept under these conditions for 1 week before experiment. All surgeries were performed under sodium pentobarbital anesthesia, and all efforts were made to minimize suffering. The 20 nude mice were administered streptozotocin (STZ, Sigma) intraperitoneally at dose of 40 mg/kg for 5 days. Mice with a blood-glucose level ≥ 300 mg/dL sustained for 7 days starting the seventh day after the last STZ injection were considered as diabetes. Fifteen diabetic nude mice were evenly divided into three groups: diabetic mice treated with block only (control), diabetic mice treated with hMSC blocks (hMSCs group), and diabetic mice treated with IPC blocks (IPCs group). In anesthetized mice, individual cell blocks consisting of 3 × 10^6^ hMSCs or IPCs were implanted into the renal sub-capsular space.

After surgery, the animals were placed on a heating pad maintained at 38.5°C, and their breathing function was monitored every 30 minutes until they were completely awake. The blood samples were obtained from the tail vein and measured for blood glucose levels every 3 days. Twenty-one days after transplantation, mice were sacrificed with the heart perfusion of 4% phosphate-buffered formalin for 10 min under anesthesia. Then the kidneys were separated, fixed in 10% formalin, embedded in paraffin, and sectioned (5 μm thick sections) for histological studies. Hematoxylin and eosin (HE) staining was performed to examine the morphology of the transplanted cells.

### Immunofluorescence staining

hMSCs or IPCs were fixed in 4% paraformaldehyde, treated with 3% H_2_O_2_ and blocked with horse serum, then incubated with monoclonal antibodies against specific CD markers, insulin, PDX-1 or c-peptide (diluted 1:1000) and then incubated with the relevant IgG conjugated with fluorescence CY3 or FITC (1:200). The cell clusters from three independent inductions were digested and re-cultured for 24 h in a lysine coated 24-well plate before fixation. Cells were then stained with anti-human c-peptide and PDX-1 antibodies, followed by incubation with the relevant secondary antibodies labeled with CY3 or FITC.

For staining of tissue sections, slides were deparaffinized in xylene and rehydrated in graded alcohol solutions. They were then treated with retrieval solution (Dako, Carpinteria, CA) and incubated with the primary antibodies including BrdU, PCNA, insulin and PDX-1 (1:1000, Cell Signaling) and then with the secondary antibodies of CY3 or FITC. Fluorescence signals were then detected by laser scanning confocal microscopy (Olympus FV500, Japan).

### Apoptosis assay

Celllar apoptosis was analyzed using an Annexin V-EGFP/PI kit (Nanjing KeyGEN Biotech. Nanjing, China). Briefly, cell pellets were re-suspended in binding buffer followed by incubation with 5 ml of Annexin V (conjugated with FITC) and PI in the dark for 10 min. Fluorescence was analyzed by the FACS Calibur. Cells positive for Annexin V-FITC and negative for PI were considered apoptotic and those positive for both Annexin V-FITC and PI were considered necrotic.

The apoptosis of transplanted cell-blocks in nude mice was analyzed by TUNEL assay using a ApopTag Peroxidase In Situ Apoptosis Detection Kit (Chemicon, CA) as previously described [26]. Under the microscope, the cells with dark-brown nuclei were positive and counted in 10 randomly selected fields per nude mice with total of 5 nude mice in each group. Results were presented as TUNEL positive cells per 100 cells.

### Statistical analysis

Data were presented as mean ± S.D. Comparisons were performed by One-way ANOVA for the different groups, followed by Tukey's test in pairwise repetitive comparisons with Origin 7.5 software. Statistical significance was considered as p<0.05.

## Results

### Morphological and phenotypical characterization of cultured hMSCs

**Fig 1:**
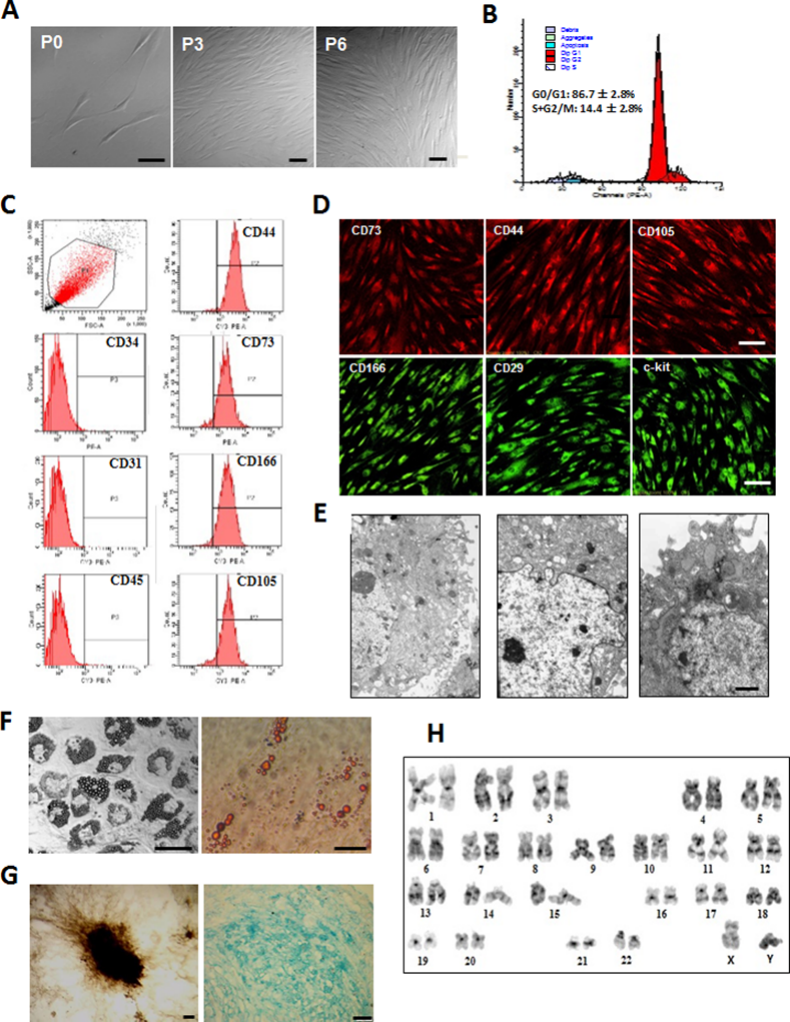
The morphological and phenotypical characteristics of cultured human bone marrow mesenchymal stem cells (hMSCs). Human bone marrow samples were obtained from healthy volunteers by lumbar puncture. Bone mononuclear cells were isolated from human bone marrow samples by density gradient centrifugation in a Percoll solution (1.073 g/ml) and cultured in L-DMEM with 10% fetal bovine serum. Eight to twelve days later, individual colonies were selected and subcultured. The morphology of cultured cells was observed and the 5^th^ passages (P5) cells were used to identified the phenotypical characteristics. A: The morphological features of cultured hMSCs at 5th days, the 3rd and 6th passages. B: Cell cycle analysis revealed that most of P5 hMSCs were in the G0/G1 phase (quiescent phase, 86.7 ± 2.8%) with a small population of cells in the S+G2/M phase (active proliferative phase, 14.4 ± 2.8%). C: Flow cytometry analysis disclosed that P5 hMSCs were positive for CD44, CD73, CD166 and CD105, but negative for CD34, CD31, and CD45. D: Immunofluorescence staining showed almost all of P5 hMSCs expressed the antigens of CD73, CD44, CD105, CD166, CD29 and c-kit. E: Under transmission electron microscope, hMSCs exhibited abundant microvilli on cell surface (upper), the irregular shape of nucleus with plentiful euchromatin (middle) and abundant organelles and glycogen granules in cytoplasm (bottom). To detect multi-lineage differentiation potential, P5 hMSCs were cultured in adipogenic, osteogenic, or chondrogenic medium for 2–4 weeks. F: After the induction, lipid droplets in cytoplasm and Oil-Red-positive adipogenic cells were observed. G: The osteogenic differentiation of hMSCs was demonstrated by mineral deposits formed and positive Von Kossa staining (left). The chondrogenic differentiation was reflected by positive Alcian blue staining (right). H: Karyotype analysis revealed the normal karyotype of P5 hMSCs. Scale bars: 50 μm for A, D, F, G and 1 μm for E.

After reaching 80% confluence, P1 hMSCs were trypsinized and passaged every 4–5 days for 9–12 passages without morphologic alteration. Typical primary or passaged hMSCs displayed fibroblast-like shape, and homogenous and vortex-like growth in monolayers (Fig 1A).

As shown in Fig 1B, cell cycle analysis revealed that most of the hMSCs at passage 5 (P5) were in the G_0_/G_1_ phase (quiescent phase, 86.7 ± 2.8%) with a small population of cells in the S+G_2_/M phase (active proliferative phase, 14.4 ± 2.8%). The surface antigens on the P5 hMSCs were analyzed by flow cytometry. hMSCs were positive for CD105, CD73 (mesenchymal stem cell markers), CD166 and CD44 (mesenchymal cell marker), but negative for CD34 (hematopoietic stem/progenitor cell marker), CD31 (endothelial cell marker), and CD45 (leukocyte cell marker) (Fig 1C). Immunofluorescence staining confirmed these results and showed that almost all of hMSCs expressed CD29 and c-kit (Fig 1D). Under the transmission electron microscope, hMSCs exhibited abundant microvilli on the cell surface, irregularly shaped nuclei with plentiful euchromatin and abundant organelles and glycogen granules in the cytoplasm (Fig 1E). We also evaluated the adipogenic, osteogenic, and chondrogenic differentiation potentials of hMSCs. After the cells were cultured in adipogenic medium for 14 days, lipid droplets in the cytoplasm and Oil-Red-positive adipogenic cells were observed (Fig 1F). hMSCs cultured in osteogenic medium formed mineral deposits as shown by positive Von Kossa staining (Fig 1G, left). Similarly, after induction for 21 days in chondrogenic medium, Alcian Blue staining showed that hMSCs expressed proteoglycan, which is indicative of chondrogenic differentiation (Fig 1G, right). P5 hMScs also show the normal karyotype (46 XY, Fig 1H). These results demonstrate the relative homogeneity of the hMSCs, which also exhibited multi-lineage differentiation potentials and highly active protein synthesis.

### Morphological changes and validation of IPCs derived from hMSCs

**Fig 2:**
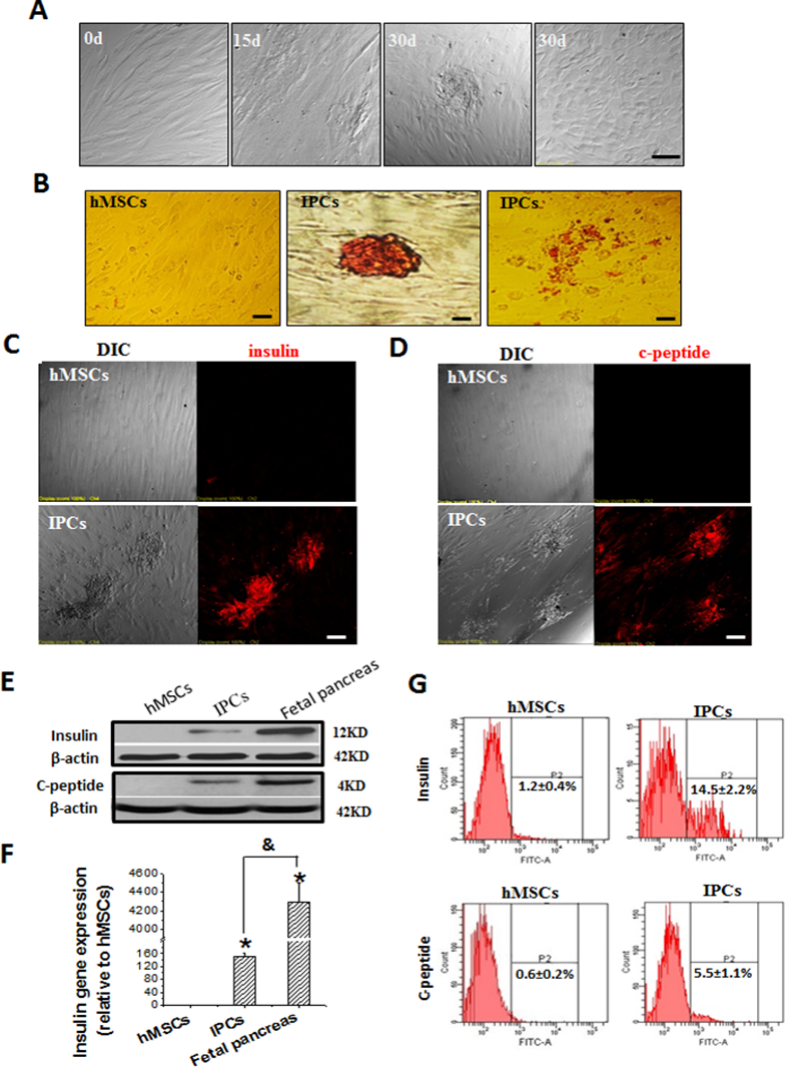
HMSCs differentiated into insulin-producing cells (IPCs). hMSCs at passage 5 (P5) were induced to differentiate into IPCs by a 30-day protocol. Briefly, hMSCs were induced in high glucose (23.3 mmol/L)-DMEM with 5% FBS for 15 days (referred to as HD-hMSCs); then cultured in L-DMEM medium containing 5% FBS and 20 μmol/L nicotinamide for 7 days; at last, Exendix-4 at 10 μmol/L was added into the medium for another 7-day incubation. After the 30-day induction, cells were fixed or harvested for characterization. A: During the 15 days of induction, small cell aggregates were formed, and during continued culture, more islet-like clusters and round epithelial-like cells appeared. B: The islet-like clusters (middle) and round epithelial-like cells (right) of differentiated hMSCs were positive for Dithizone staining (red color), while the undifferentiated hMSCs were negative. C and D: Immunofluorescence staining showed that islet-like clusters expressed both insulin (C) and c-peptide (D). E: Western blot assay further confirmed the expression of insulin and c-peptide proteins in the induced cells, the fetal pancreas from 6-month-old aborted fetus was used as control. F: The insulin gene expression was evaluated by real-time PCR in hMSCs and IPCs, for which the fetal pancreas was used as positive control. G: Flow cytometry analysis demonstrated that there was 14.5 ± 2.2% of insulin-positive cells and 5.5 ± 1.1% of c-peptide positive cells in the induced cells from P5 hMSCs. All in vitro data were obtained from at least 3 independent experiments. Data are presented as means ± SD (* P<0.05 compared to hMSCs; ^&^ P<0.05 compared between hMSCs and fetal pancreas). Scale bars: 50 μm for A-D.

P5 hMSCs were induced to differentiate into IPCs by a 3-step, 30-day protocol. During the 15 days of step 1, small cell aggregates were formed. During continued culture, round epithelial-like cells appeared and more islet-like clusters were observed (Fig 2A). Taking advantage of the fact that DTZ staining can be used to specifically recognize insulin granules in β-cells, we found that the “differentiated” islet-like clusters and round epithelial-like cells had insulin granule structure (Fig 2B), while the undifferentiated cells were negative for DTZ staining. C-peptide is released when pro-insulin is cleaved to insulin and as such, it has been considered as the marker of insulin secretion. Immunofluorescence staining showed that islet-like clusters expressed both insulin (Fig 2C) and c-peptide (Fig 2D). Western blot assay further confirmed the expression of insulin and c-peptide proteins in the induced cells, even though their expression levels were much lower than that of positive control fetal pancreas (Fig 2E). In addition, the expression of the insulin gene was also detected in the induced cells by real-time PCR; however, its expression was significantly lower than that in fetal pancreatic control cells (Fig 2F). Moreover, flow cytometry analysis demonstrated that there was 14.5 ± 2.2% of insulin-positive cells and 5.5 ± 1.1% of c-peptide positive cells in the induced cells from P5 hMSCs (Fig 2G).

### Gene expression and insulin-releasing functions of IPCs *in vitro*

**Fig 3:**
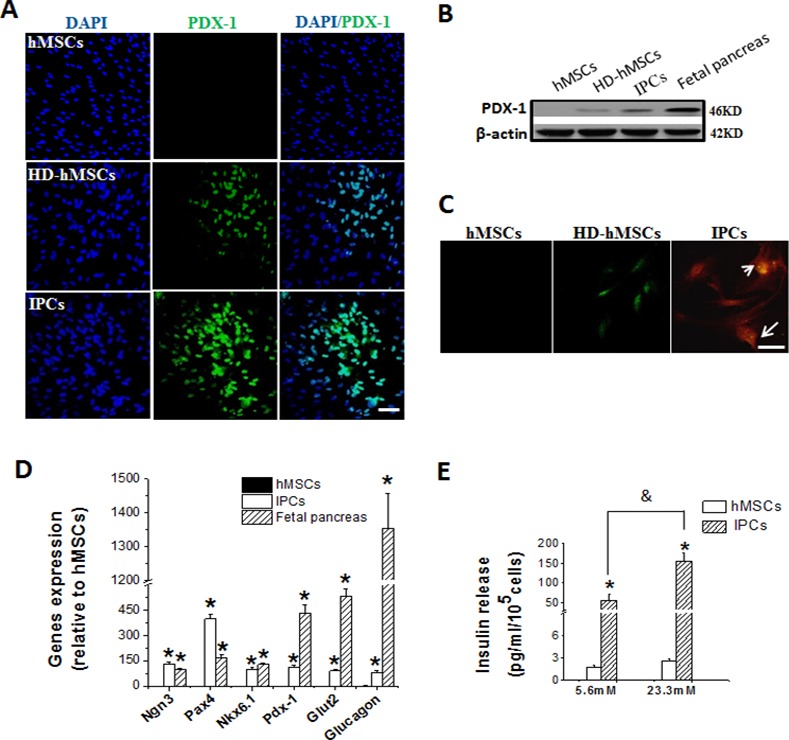
Gene expression and insulin-releasing functions of IPCs *in vitro*. hMSCs were induced and treated under the same condition described in Fig 2. A: Immunofluorescence staining for PDX-1 (green) and nuclear staining with DAPI (blue) on hMSCs, HD-MSCs and IPCs. The PDX-1 staining in hMSCs was negative, while HD-MSCs and IPCs expressed PDX-1 in nuclei (green). B: Western blot showed that PDX-1 was activated in the induced cells (HD-hMSCs) and the expression of PDX-1 was significantly elevated in IPCs. C: The cell aggregates/clusters from HD-hMSCs and IPCs groups were picked, digested and re-cultured to get the monolayer cells, and the co-staining for PDX-1 (green) and c-peptide (red) was performed on the monolayer cells. hMSCs were negative for both PDX-1 and c-peptide, and HD-hMSCs only expressed the PDX-1, while the monolayer cells from IPCs co-expressed PDX-1 and c-peptide. D: Expression levels of Pdx1, Ngn3, Pax4, Nkx6.1, Glut2 and glucagon genes by quantitative real-time PCR test. The IPCs expressed Pdx1, Ngn3, Pax4, Nkx6.1, Glut2 and Glucagon, similar to the expressing of fetal pancreas. E: The human insulin secreted by IPCs in response to glucose stimulation was examined by ELISA assay. Insulin released amount was calculated by insulin secreted in the culture medium for 2 h after 5.6mM and 23.3mM glucose stimulation. Compared to hMSCs, IPCs show the insulin secretion in a glucose dose-dependent manner. Data are presented as means ± SD from the 3 independent experiments (*P<0.05 compared to hMSCs; ^&^*P*<0.05 compared between 5.6mM and 23.3mM glucose groups). Scale bars: 50μm for A and C.

The PDX-1 gene plays an important role in the activation of β-cell-specific insulin expression and in the regulation of β-cell differentiation [25]. Immunofluorescence staining and western blot showed that after induction for 15 days in the high glucose condition, PDX-1 was activated in the induced cells (HD-hMSCs) and that after a continual induction of 30 days, the expression of PDX-1 was significantly elevated (Fig 3A and 3B). Furthermore, to determine whether PDX-1 positive cells actually secreted insulin and to avoid the non-specific immunofluorescence staining on the cell that are aggregated, the cell aggregates/clusters from HD-hMSCs and IPCs groups, were picked, digested by trpsin and re-cultured for 24 h to get the monolayer cells, the co-staining for PDX-1 and c-peptide was performed on those monolayer cells. As shown in Fig 2C, monolayer cells from HD-hMSCs only expressed the PDX-1, while the monolayer cells from IPCs co-expressed PDX-1 and c-peptide. Quantitative real-time PCR results demonstrated that the induced cells expressed Pdx1, Ngn3, Pax4, Nkx6.1, Glut2 and Glucagon, similar to the fetal pancreas of positive control (Fig 3D). However, the induced cells expressed significantly higher Pax4, and lower Pdx-1, insulin, Glut2 and glucagon than fetal pancreatic islets (all *P* < 0.01).

The human insulin secreted by IPCs in response to glucose stimulation in the culture medium was examined by ELISA assay (Fig 3E). The amount of human insulin released by IPCs of 10^5^ cells in response to 5.6 mM or 23.3 mM glucose was 56.4 ± 16.5 pg/ml and 156.2 ± 20.8 pg/ml respectively, while in the undifferentiated hMSCs, the insulin amount was less than 2.5 pg/ml. These results suggested that compared to hMSCs, which exhibit no obvious insulin secretion, IPCs do secret insulin in a glucose dose-dependent manner (*P* < 0.05).

### Measurement of L-type Ca^2+^ channels during the differentiation process

**Fig 4:**
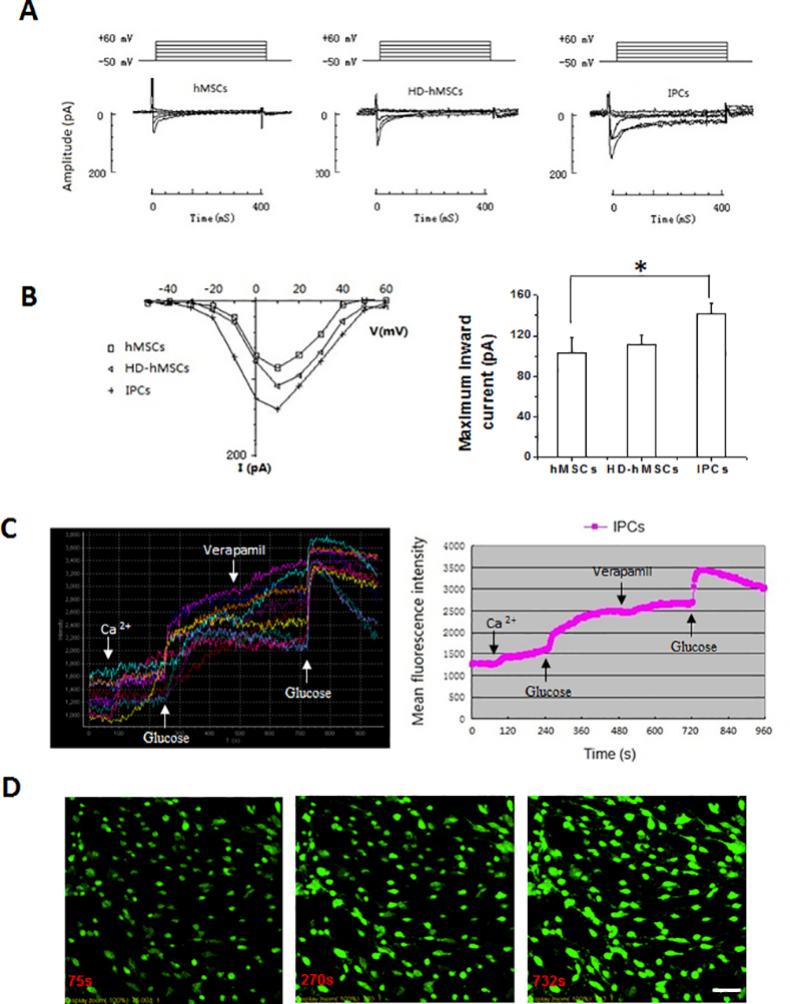
Measurement of L-type Ca^2+^ channels during differentiation process and intracellular Ca^2+^ concentration in IPCs in response to glucose stimulation. The whole-cell patch-clamp experiments were performed on hMSCs, HD-MSCs and IPCs to determine the amount and functional change of L-type Ca^2+^ channel during the differentiation process. Nifedipine at 10 μM suppressed the inward currents. Nifedipine-sensitive inward Ca^2+^ currents (I_Ca.L_) were recorded by 300-ms voltage step between +10 and +60 mV from a holding potential of –50 mV (to inactivate INa). A: The I_Ca.L_ was recorded among the hMSCs, HD-MSCs and IPCs. B: The I-V relationship of I_Ca.L_ displayed the maximum current peak appeared at 10 mV in hMSCs, HD-MSCs and IPCs (left), and the mean peak current amplitudes in IPCs is higher than that in hMSCs or HD-MSCs (right, *P<0.05 compared to hMSCs). C: The changes of intracellular Ca^2+^ concentration in IPCs in response to glucose stimulation were measured by labeling with Fluo-3/AM under laser confocal scanning microscopy. The results were expressed as respective fluorescence intensity of 10 of IPCs (left) and average fluorescence intensity of 30 of IPCs (right). After stimulated with 30mmol/L glucose at 240 s, the intracellular Ca^2+^ concentration rapidly increased in IPCs due to significant Ca^2+^ influx when extracellular Ca^2+^ existing. When Ca^2+^ channels were blocked by 25 mg/ml of Verapamil at 480 s and then stimulated with 3.5 mM glucose at 720 s, intracellular Ca^2+^ rapidly increased again in IPCs due to the release of Ca^2+^ from calcium stores. D: The images of intracellular Ca^2+^ before (left) and after 2 times of glucose stimulation (middle and right) in IPCs, reflected by green fluorescence. Scale bars: 50μm for D.

It is reported that L-type Ca^2+^ channels are involved in the regulation of MSC differentiation and play a crucial role in stimulus-secretion coupling in human pancreatic β-cells [27]. Patch-clamp experiments were performed on hMSCs, HD-MSCs and IPCs to define whether there is the amount and functional change of L-type Ca^2+^ channel in the differentiation process. Fig 4A illustrated the nifedipine-sensitive inward Ca^2+^ currents (I_Ca.L_) existing in hMSCs, HD-MSCs and IPCs. Moreover, of the hMSCs, HD-MSCs and IPCs, about 18% (5 out of 28), 25% (9/36), and 43% (13/30) had their ICa.L recorded and the maximum I_Ca.L_ were seen at +10 mV, with mean peak current amplitudes of -103.0 ± 15.3, -111.8 ± 9.0 and -141.7 ± 10.8 pA, respectively (Fig 4B). Compared with hMSCs, the amount and mean peak current of L-type Ca^2+^ channels were significantly increased in IPCs (*P* < 0.05), suggesting that IPCs had more mature L-type Ca^2+^ channels.

### Measurement of intracellular Ca^2+^ concentration in IPCs in response to glucose stimulation

Insulin release from pancreatic β-cells is a Ca^2+^-dependent process. In response to glucose, there is a Ca^2+^ influx resulting in an increased Ca^2+^ concentration in the cytoplasm that subsequently initiates the release of insulin [27]. Therefore, the intracellular Ca^2+^ concentration changes in IPCs in response to glucose stimulation were measured by labeling with Fluo-3/AM and the cells were visualized under laser confocal scanning microscopy. As shown in Fig 4C and 4D, the level of intracellular Ca^2+^ at the resting stage was examined first with the average fluorescence intensity (AFI) of 30 IPCs recorded as 1269.4 ± 222.8. The addition of 15 μM of extracellular Ca^2+^ to the observation chamber at 60 s did not cause obvious changes in the intracellular Ca^2+^ concentration; however, after stimulation with 30 mM glucose at 240 s, the intracellular Ca^2+^ concentration rapidly increased in IPCs due to significant Ca^2+^ influx, and as such, the amplification of AFI was 1004.8 ± 323.1. Moreover, when Ca^2+^ channels were blocked by 25 mg/ml of Verapamil at 480 s and then stimulated with 3.5 mM glucose at 720 s, intracellular Ca^2+^ concentration rapidly increased again due to the release of Ca^2+^ from calcium stores and the amplification of AFI was 878.8 ± 254.4. These results demonstrated that glucose stimulation-induced intracellular Ca^2+^ changes were associated with insulin release in differentiated IPCs, which is a similar pattern of intracellular Ca^2+^ changes than those observed in mature pancreatic β-cells.

### Proliferation and apoptosis of IPCs

**Fig 5:**
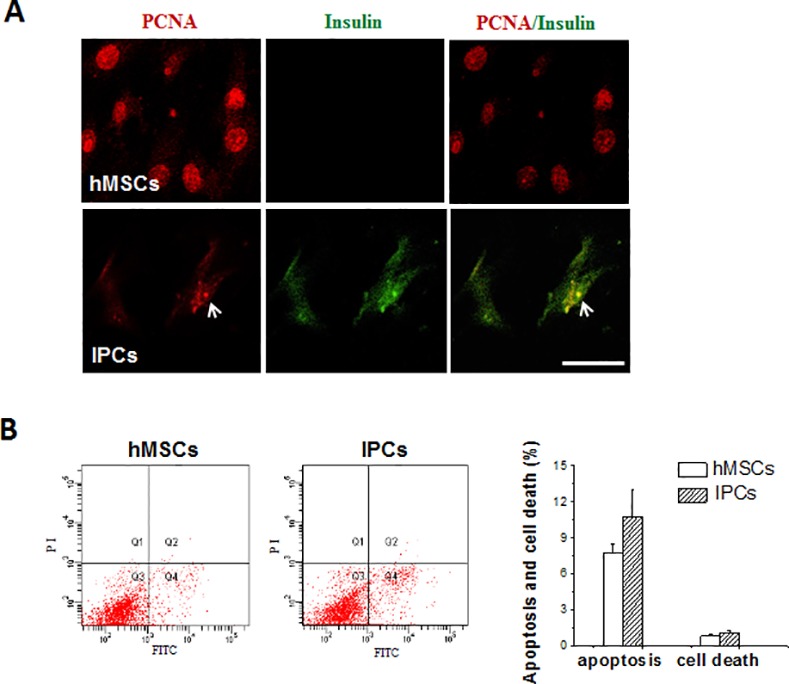
Proliferation and apoptosis of IPCs. hMSCs were induced and treated under the same condition described in Fig 2. A: Immunofluorescence staining for PCNA (red) and insulin (green) on hMSCs and IPCs (400×). The PCNA, not insulin was strongly expressed in undifferentiated hMSCs. While a portion of IPCs expressed both PCNA and insulin. B: The IPCs did not show significant apoptosis and necrosis compared with hMSCs examined by Annexin V-EGFP/PI apoptosis kit. Scale bars: 50μm for A.

To evaluate the survival of differentiated IPCs, double immunofluorescence staining of PCNA and insulin and flow cytometry assay of the apoptosis marker Annexin V-EGFP/PI were performed. As shown in Fig 5A, hMSCs were positive for PCNA and negative for insulin. A portion of IPCs show the co-expression of PCNA and insulin, however, compared with undifferentiated hMSCs, the expression of PCNA in IPCs was weaker. The apoptotic rates in hMSCs and IPCs were 7.7 ± 0.8% and 10.7 ± 2.3% respectively, while the necrosis rates were 0.8 ± 0.1% and 1.1 ± 0.2% respectively. The IPCs did not show significant apoptosis and necrosis compared with hMSCs (*P*>0.05, Fig 5B).

### Transplantation of IPCs into STZ-induced diabetic nude mice *in vivo*

**Fig 6:**
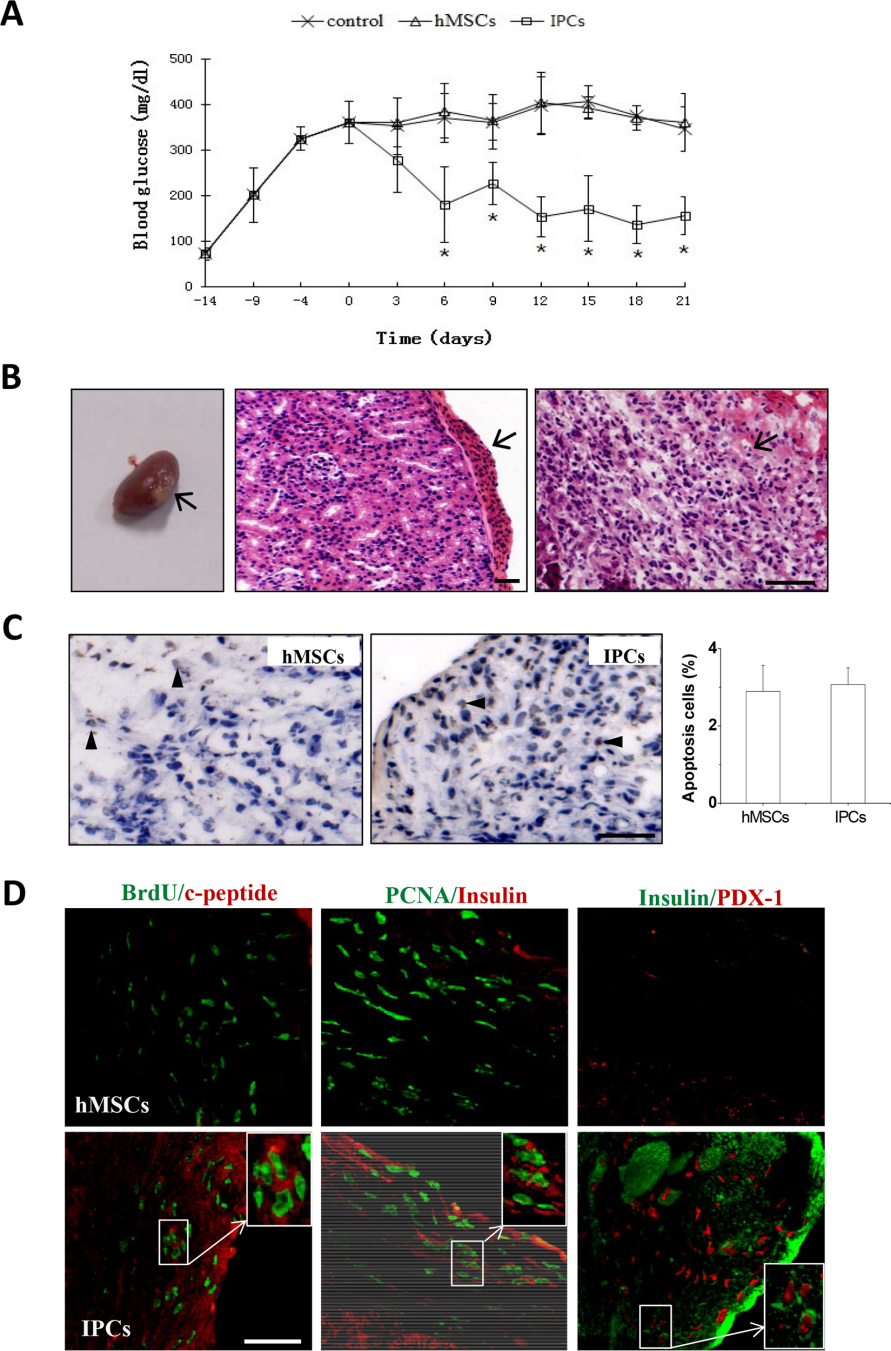
Transplantation of IPCs into STZ-treated diabetic nude mice *in vivo*. IPCs or hMSCs were transplanted into renal capsule of STZ-induced type 1 diabetic male BALB/C nude mice. Blood glucose was measured every 3 days for 21 days. Twenty-one days after transplantation, mice were sacrificed by heart perfusion of 4% phosphate-buffered formalin and the kidneys were isolated, fixed in 10% formalin, embedded and sectioned for histological studies. A: Blood glucose of the diabetic mice implanted with IPCs were normalized and remained euglycemic throughout the observation period of 21 days, whereas those receiving no cells or hMSCs remained hyperglycemic (*P<0.05 compared to non-transplanted or hMSCs groups, n = 5). B: Gross appearance (left) and hematoxylin and eosin staining showed the presence of transplanted IPCs under the renal capsule (arrows). C: TUNEL staining revealed that there was no obvious apoptosis in the transplanted hMSCs or IPCs under the renal capsule. D: Immunofluorescence staining indicated that IPCs expressed insulin, PDX-1 and c-peptide under the renal capsule. Scale bars: 50μm for B-D.

Blood glucose levels of the diabetic mice implanted with IPCs were normalized within 6 days and the mice remained euglycemic throughout the observation period of 21 days, whereas those receiving no cells or hMSCs remained hyperglycemic (Fig 6A). At the end of the observation period, histological examination of the extracted kidneys revealed the presence of transplanted cell blocks under the renal capsule (Fig 6B). TUNEL assay showed no obvious apoptosis in the transplanted hMSCs or IPCs (less than 3%, Fig 6C), neither was there a further increase compared to the apoptosis that had been seen *in vitro*. Immunofluorescence staining revealed that transplanted IPCs labeled with BrdU secreted insulin *in vivo*, which was demonstrated by the positive staining for c-peptide. Some IPCs could also regrow due to the co-expression of PCNA and insulin. Mature IPCs *in vivo* were identified by co-expression of PDX-1 and insulin. It was observed that some of the transplanted cells were still differentiated and not immature as demonstrated by positive staining for PDX-1 and negative for insulin. As seen in the control, the transplanted hMSCs just expressed the PCNA, and not insulin or PDX-1 (Fig 6D).

## Discussion

MSCs derived from human adult bone marrow of healthy donors were selected based on their ability to adhere to plastic culture dishes, which were consistent with other reports [20]. To get more uniform hMSCs, individual colonies were selected and expanded for 8–12 days after the original seeding of bone mononuclear cells. The cells with spindle-shaped morphology were negative for CD34, CD31, and CD45 and positive for CD44, CD73, CD166, CD29, CD105, and c-kit (Fig 1C and 1D). The cell cycle analysis showed that most of the cells were in the quiescent phase (G_0_/G_1_ phase). In addition, the isolated cells exhibited ultrastructure characteristics of active protein synthesis and normal karyotype (Fig 1H), as well as their capacity to undergo adipogenic, chondrogenic, and osteogenic differentiation (Fig 1B and 1E–1G). These results fully testified that highly homogenous and pluripotent hMSCs were obtained.

Despite the fact that various protocols exist to induce hMSCs to differentiate into IPCs *in vitro*, these induction strategies need to be modified due to their complicated processes and lower efficiencies [3, 6, 21]. In our three-stage induction protocol, we simplified the inducer agents and extended the induction period to obtain better differentiation, while slightly increasing the efficiency of induction. High glucose has been considered as a potent inducer for pancreatic islet differentiation [28], and so DMEM with 23.3 mmol/L glucose was used at the first stage to initiate differentiation. In fact, at the end of this stage, cells had formed small cell aggregates (Fig 2A) and expressed activated PDX-1 (Fig 3A–3C), which is one of the key transcription factors in pancreatic β-cell differentiation and maturation [29]. Extended treatment with 20–30 mmol/L glucose has been reported to inhibit β-cell differentiation [17]; as such, at stage 2 of induction, the glucose concentration was reduced to 5.6 mmol/L and nicotinamide was added to prevent differentiated cells from dying or differentiating into other cell types [30]. Due to its function on stimulation of β-cells replication and inhibition of β-cells apoptosis, Exendin-4 was added during stage 3 [20]. After the three-stage induction, islet-like clusters were formed (Fig 2A) and the induced cells showed increased expression of a suite of pancreatic genes including Insulin, Glut2, PDX-1, Ngn3, Pax4, Nkx6.1 and Glucagon (Figs 2F and 3D). The successful differentiation of hMSCs into IPCs by this three-stage induction is furthered ascertained by the fact that differentiated cells were positive for DTZ staining (Fig 2B), expressed insulin and c-peptide (Fig 3C–3E), and secreted insulin in a glucose-regulated manner (Fig 3E). Moreover, our methodology slightly improved the efficiency of hMSC induction into IPCs *in vitro*: ∼15% of insulin-positive cells and ∼6% of c-peptide-positive cells (Fig 2G), compared to the previously reported observation of only 5% insulin-expressing cells derived from human MSCs [31]. Distressingly, previous publications mistook the fact that the induced cells could uptake insulin from the media (supplied with the N2 or/and B27 containing additional insulin) as a positive identification of IPCs [32, 33]. Hence, in the present study, only media without insulin was used to ensure that any insulin secreted would be produce from the IPCs themselves.

It is considered that undifferentiated hMSCs are non-excitable cells. Ca^2+^ signaling pathways are expected to play a key role in the transformation to excitable cell types such as neurons, muscles, and β-cells [34]. One of the main sources of Ca^2+^-generated signals is Ca^2+^ entry across the plasma membrane via voltage-operated Ca^2+^ channels (VOCCs) [35]. Gui et al. reported that among many kinds of the VOCCs, only L-type Ca ^2+^ channels were detected in 15% of undifferentiated hMSCs [24, 36]. Consistent with this, we recorded about 18% undifferentiated hMSCs having a nifedipine-sensitive L-type Ca ^2+^ current (I_Ca.L_). In contrast, after the three-stage induction, 43% of IPCs were recorded to have I_Ca.L_, which strength was also significantly increased (Fig 4A and 4B). This observation demonstrates that hMSCs could be transformed into excitable IPCs, which use a much more mature L-type Ca^2+^ channels for cell functions.

It is well known that L-type Ca^2+^ channels play crucial roles in stimulus-secretion coupling in human pancreatic β-cells. In fact, insulin release from pancreatic β-cells is a Ca^2+^-dependent process. Glucose stimulation causes an increase of intracellular ATP, closure of ATP-sensitive potassium channels, subsequent opening of L-type Ca ^2+^ channels and, thus, Ca^2+^ influx. The increased intracellular Ca^2+^ concentration activates Ca^2+^ release from the endoplasmic reticulum and sarcoplasmic reticulum. This further increment in the Ca^2+^ concentration in the cytoplasm initiates the secretion of insulin [27]. In line with this process of insulin secretion, IPCs undergo intracellular Ca^2+^ changes in response to glucose stimulation similar to that in pancreatic β-cells (Fig 4C and 4D).

The blood glucose levels of the diabetic mice were normalized within 6 days after transplantation of IPCs and remained in the normal range throughout the observation period of 21 days, while mice treated with no cells or undifferentiated hMSCs remained hyperglycemic (Fig 6A). Consistent with the *in vitro* study, transplanted cells in the renal subcapsular space stained positive for insulin and c-peptide (Fig 6D); moreover, no obvious positive TUNEL staining was observed (Fig 6C), suggesting that the transplanted IPCs could survive *in vivo* and secrete functional insulin. All these findings *in vivo* strongly indicate that hMSCs-derived IPCs could quickly and effectively control hyperglycemia in diabetes.

The efficiency of differentiation of hMSCs into IPCs is critical for the supplement of enough cells for clinical transplantation and production of sufficient insulin for glycemic control. It has been proven that a large number of cells (10 million/mouse, equivalent to 1000 functional islets) in a mouse-to-mouse transplantation is needed to correct hyperglycemia *in vivo* when either the differentiation efficiency or the insulin content was low [21, 31]. In our induction strategy, about 15% of differentiated hMSCs express insulin *in vitro* (Fig 2G), which will not be enough to correct hyperglycemia of the transplanted mice when using 10^6^~10^7^ differentiated cells/mouse *in vivo*. However, our findings indicate that 3 × 10^6^ differentiated cells (containing about 5 × 10^5^ IPCs) could efficiently alleviate the hyperglycemia *in vivo*. As such, we speculate that immature cells in the transplanted cell blocks complete their differentiation and maturation *in vivo*, which is possibly associated with the 3-dimensional structure supplied by the mixture of fibrinogen and thrombin, as well as the growth factors and hyperglycemia in the microenvironment. This notion is supported by previous studies [37, 38] that demonstrated that 3-dimensional support, supplied by Matrigel, promoted ESC or MSC differentiation to IPCs. Karnieli et al. also observed that PDX-1-expressing MSCs further differentiated after cell transplantation *in vivo* as indicated by the expression of NEUROD1, which is a key transcription factor in differentiated β-cells [16].

Interestingly, IPCs expressed PCNA, which is a marker of cell proliferation. Although the expression of PCNA in IPCs was not as strong as in undifferentiated hMSCs, it seemed to have contributed to the efficiency of IPCs in controlling hyperglycemia *in vivo*. In fact, PCNA was found to co-localize with insulin in specific areas of differentiated cells (PCNA^+^/Ins^+^ cells) *in vitro* (Fig 5A) and *in vivo* after cell transplantation (Fig 6D). This observation is interesting in light of the evidence that stem cells or progenitor cells exit from the cell cycle upon differentiation [39]. Furthermore, human pancreatic islet cells are believed to be terminally differentiated cells unable to proliferate or regrow. This discrepancy might be explained with the notion that differentiated cells co-expressing insulin and PCNA could simply constitute a transient intermediate stage that subsequently stops proliferation and undergoes further maturation in a manner similar to the transient amplifying progenitor cells described in epidermal development and differentiation [40].

In conclusion, our study demonstrates that human bone marrow-derived MSCs can differentiate into insulin-producing cells *in vitro*, which effectively function both *in vitro* and *in vivo*. All these findings support the potential of hMSCs as a promising tool for the treatment of insulin-dependent diabetes. Differentiation of autologous MSCs into IPCs could provide dramatic clinical benefits for diabetic patients. To facilitate this goal, the length of the IPC functionality *in vivo*, the determination and generation of the necessary cell numbers for transplantation, as well as the identification of the optimal site for implantation need to be further characterized.
